# Whole blood RNA signatures in tuberculosis patients receiving H56:IC31 vaccine as adjunctive therapy

**DOI:** 10.3389/fimmu.2024.1350593

**Published:** 2024-02-16

**Authors:** Noelia Alonso-Rodríguez, Eleonora Vianello, Suzanne van Veen, Synne Jenum, Kristian Tonby, Rosalie van Riessen, Xiaoran Lai, Rasmus Mortensen, Tom H. M. Ottenhoff, Anne Ma Dyrhol-Riise

**Affiliations:** ^1^ Department of Infectious Diseases, Oslo University Hospital, Oslo, Norway; ^2^ Department of Infectious Diseases, Leiden University Medical Center, Leiden, Netherlands; ^3^ Institute of Clinical Medicine, University of Oslo, Oslo, Norway; ^4^ Oslo Centre for Biostatistics and Epidemiology, Faculty of Medicine, University of Oslo, Oslo, Norway; ^5^ Deptartment of Infectious Disease Immunology, Statens Serum Institut, Copenhagen, Denmark

**Keywords:** tuberculosis, H56:IC31 vaccine, host directed therapy, transcriptomics, RNA signature

## Abstract

**Introduction:**

Therapeutic vaccination in tuberculosis (TB) represents a Host Directed Therapy strategy which enhances immune responses in order to improve clinical outcomes and shorten TB treatment. Previously, we have shown that the subunit H56:IC31 vaccine induced both humoral and cellular immune responses when administered to TB patients adjunctive to standard TB treatment (TBCOX2 study, NCT02503839). Here we present the longitudinal whole blood gene expression patterns in H56:IC31 vaccinated TB patients compared to controls receiving standard TB treatment only.

**Methods:**

The H56:IC31 group (N=11) and Control group (N=7) underwent first-line TB treatment for 182 days. The H56:IC31 group received 5 micrograms of the H56:IC31 vaccine (Statens Serum Institut; SSI, Valneva Austria GmbH) intramuscularly at day 84 and day 140. Total RNA was extracted from whole blood samples collected in PAXgene tubes on days 0, 84, 98, 140, 154, 182 and 238. The expression level of 183 immune-related genes was measured by high-throughput microfluidic qPCR (Biomark HD system, Standard BioTools).

**Results:**

The targeted gene expression profiling unveiled the upregulation of modules such as interferon (IFN) signalling genes, pattern recognition receptors and small nucleotide guanosine triphosphate (GTP)-ases in the vaccinated group compared to controls two weeks after administration of the first H56:IC31 vaccine. Additionally, the longitudinal analysis of the Adolescent Cohort Study-Correlation of Risk (ACS-COR) signature showed a progressive downregulation in both study arms towards the end of TB treatment, in congruence with reported treatment responses and clinical improvements. Still, two months after the end of TB treatment, vaccinated patients, and especially those developing both cellular and humoral vaccine responses, showed a lower expression of the ACS-COR genes compared to controls.

**Discussion:**

Our data report gene expression patterns following H56:IC31 vaccination which might be interpreted as a lower risk of relapse in therapeutically vaccinated patients. Further studies are needed to conclude if these gene expression patterns could be used as prognostic biosignatures for therapeutic TB vaccine responses.

## Introduction

Tuberculosis (TB) remains one of the deadliest infectious diseases worldwide and the emergence of multi drug resistant TB is a global concern (1). The effects of COVID-19 on global TB control efforts have been catastrophic, reversing years of progress in the TB epidemic control (1). Thus, new effective TB vaccines are urgently needed to achieve the goal of eradicating TB as a global public health problem (2, 3).

Whilst preventive TB vaccination aims to prevent TB disease, the purpose of therapeutic TB vaccination is to reshape host immunity in order to improve outcomes, shorten treatment duration, and/or prevent relapse by establishing long-lasting specific immunological memory (4, 5). Several therapeutic TB vaccine candidates are currently in the pipeline (6), showing promising results in mice and non-human primates (7). The subunit H56:IC31 vaccine was conceptualized to target different stages of Mtb infection (8). It consists of a recombinant fusion protein (H56) entailing the Mtb antigens Ag85B, secreted early after the infection, ESAT-6, constitutively expressed, and Rv2660c, expressed mainly in Mtb latent forms (8). The H56:IC31 vaccine has been tested for safety and immunogenicity in different clinical trials (9–12) (https://newtbvaccines.org/vaccine/h56ic31/). In our previous first-in-human randomized clinical trial (NCT02503839, TBCOX2), we demonstrated the safety and immunogenicity of adjunct administration of the H56:IC31 vaccine during TB disease (9), suggesting a potential to serve as a therapeutic vaccine. Also, an ongoing clinical trial (NCT03512249) evaluates the efficacy of H56:IC31 on the prevention of TB recurrence when administered at the end of TB treatment.

The development of new TB vaccines has long been hampered by the lack of immune correlates of protection. Although T cell immunity is regarded crucial for long-term protection (13–15), and a protective role of antibodies has been suggested (16–18), the lack of correlates of protection requires resource-intensive and lengthy clinical trials assessing clinical outcomes as measures of vaccine efficacy. High-throughput host transcriptional studies have been useful in understanding the molecular mechanisms of the immune responses to vaccination. Molecular biomarkers and signatures that can predict vaccine efficacy (19) has been shown for other infectious diseases such as yellow fever (20), influenza (21), Ebola (22) and pneumococcal infections (23). Such studies have also been conducted in clinical trials testing the TB vaccines AERAS-402 (24), BCG and VPM1002 (25), M72/AS01 (26) and MVA85A (27). These works have shown a plethora of vaccine-induced transcriptomic responses that reflect study design differences such as targeted demographic group, chosen time points and genomic techniques for the gene expression analysis. As a general feature, most of the TB vaccines explored in these studies were associated with early transcriptomic responses of myeloid cells and inflammation modules (25, 26). However, a low correlation between early inflammatory transcriptomic responses and later cellular IFNγ responses have also been reported in some cohorts (27), indicating heterogeneous vaccine mediated immunogenic signatures.

Host transcriptome studies have represented a step forward in the comprehension of TB as a dynamic continuum spectrum of infection (28, 29). A persistent refinement of the first published transcriptional signature composed of 393 targeted genes (30), led to more accessible and comprehensible signatures such as the Adolescent Cohort Study-Correlation of Risk (ACS-COR/Zak-16 signature) (31), RISK11 (32, 33) and RISK6 (34, 35). These transcriptomic signatures have demonstrated a high sensitivity (85-91%) and specificity (56-86%) in the diagnosis of active TB (29, 36–38), and the identification of people at high risk of developing TB within the following months (31–34). A potential to predict treatment response has also been suggested (28, 30, 31, 39–43) as RISK signature scores increase as TB progresses and retract during TB treatment (44).

In the context of the TBCOX2-trial, herein we analyse the expression of 183 preselected genes with the goal to map longitudinal vaccine-induced transcriptomic perturbations in TB patients given adjunct H56:IC31 vaccination compared to controls receiving standard TB treatment only. Further, we explored the association between gene expression and vaccine-induced immunogenicity. Last, we analysed differences in the longitudinal gene-expression pattern of genes within the ACS-COR signature between vaccinated and non-vaccinated subjects.

## Material and methods

### Study design and participants

The present study is nested within the TBCOX2 study, a randomized, open-label, controlled, four group phase I/II clinical trial including HIV negative patients with bacteriologically confirmed active TB treated with standard antimicrobial therapy for sensitive Mtb (9). See **Table 1** for clinical characteristics. For this study, only pulmonary TB cases from the two arms, H56:IC31 (N=11) and Controls (N=7) were included. The H56:IC31 group received 5 micrograms of the H56:IC31 vaccine (Statens Serum Institut; SSI, Valneva Austria GmbH) intramuscularly at day 84 and day 140 in addition to standard TB treatment for 182 days. The controls received standard TB treatment only. Blood samples were collected on days 0, 84, 98, 140, 154, 182 and 238.

**Table 1:** Baseline characteristics of Tuberculosis patients in the H56:IC31 and Control groups.

	H56:IC31											CONTROLS						
	R	R	R	R	PR	PR	PR	NR	R	R	R	C	C	C	C	C	C	C
**Patient ID**	**01-003**	**01-005**	**01-011**	**01-015**	**01-019**	**01-022**	**01-023**	**01-025**	**01-029**	**01-032**	**02-002**	**01-004**	**01-008**	**01-021**	**01-027**	**01-030**	**01-035**	**01-040**
Demography																		
**Gender**	F	M	F	M	M	M	M	F	M	M	F	M	F	F	M	M	F	F
**Age**	34	18	26	19	21	52	21	23	25	26	20	20	44	29	23	23	27	29
**Ethnicity**	C	C	A	B	B	B	B	B	A	B	B	B	B	A	B	B	A	A
Clinical Features																		
**Cough**	1	1	0	1	0	1	1	1	1	0	0	0	2	0	0	2	0	0
**Chest Pain**	1	0	0	1	1	2	0	1	0	1	0	0	2	0	0	0	0	0
**Sweating**	1	1	0	1	0	2	1	0	1	1	0	0	2	0	0	1	1	0
**Fever**	1	0	0	0	0	0	0	0	0	0	0	0	1	0	0	1	0	0
**Weight loss**	0	0	0	1	0	0	0	1	0	0	0	0	0	0	1	0	0	0
**BMI**	21.1	21	20.6	18.8	22.7	19	18	22.3	19	21.1	20.4	27.2	25	18.5	17	20	23.3	24
Radiology																		
**Cavities**	1	0	0	0	0	0	0	0	1	0	0	0	1	0	0	1	0	0
**Bilateral infiltrates**	0	0	0	0	0	0	0	0	1	1	0	0	0	0	0	0	0	0
**Infiltrates in ≥2 unilateral lobes**	0	0	0	0	0	1	0	0	0	0	0	0	0	0	0	0	0	0
**Hilary pathology**	0	0	0	0	0	0	0	1	0	0	1	0	0	0	0	0	0	0
Laboratory data																		
**CRP**	NA	7.2	0.6	224	0.7	71	8.1	36.5	19	0	2	2.8	200	0	0	59	0	6.4
**ESR**	NA	20	20	80	7	116	11	NA	52	1	19	1	88	12	4	67	5	35
**QFT-TB**	2	2	2	3	2	2	2	2	2	2	NA	2	2	2	2	2	1	2
**AFS (grading)**	3+	NEG	1+	NEG	NEG	NEG	NEG	1+	1+	1+	NEG	NEG	4+	NEG	NEG	1+	NEG	NEG

Patient ID (Patient Identification). Patients 01-003, 01-005, 01-011, 01-015, 01-019, 01-022, 01-023, 01-025, 01-029, 01-032, and 02-002 belong to the H56:IC31 group. Patients 01-004, 01-008, 01-021, 01-027, 01-030, 01-035, and 01-040, were in the Control group.

R, responder; PR, Partial Responder; NR, Non-Responder; C, Control; F, Female; M, Male; BMI, Body Mass Index; NA, data not available.

Ethnicity A, Asian; B, Black; C, Caucasian.

Clinical Features: Cough, Chest pain: 0=no; 1=no interference with activity; 2=some interference with activity; 3=significant, prevent daily activity; 4=Emergency Room visit or hospitalization; Fever, Weight loss: 0=no; 1=yes; Sweating: 0=normal; 1=mild or occasional; 2=frequent or drenching.

Radiology: X-RAY/CT scan findings: 0=no; 1=yes; QFT-TB: 1=NEG; 2= POS; 3= Intermediate. AFS: Acid-fast staining microscopy grading: NEG: negative. Positive Grade 1+, 2+, 3+, 4+. AFS: Acid Fast Stain.

QFT-TB: QuantiFERON-TB: 1=NEG; 2= POS. CRP: C-Reactive Protein (mg/L). ESR: Erythrocyte Sedimentation Rate (min/hour).

### Definition of vaccine responder and non-responder

Immunogenicity analysis was performed as previously described (9). Mtb-specific immune responses were assessed as follows: Cytokine-producing CD4 T cells (cytokine+) were determined by the IFNγ/IL-2 immuno-spot (Fluorospot) assay. Cytokine+ was defined as the sum of IFNγ and IL-2 responses to Ag85B and ESAT-6. Vaccine IgG responses were measured by ELISA quantification of anti-H56 IgG in serum.

According to vaccine-elicited cellular and humoral immune responses patients were classified as follows:

*Responders*: patients with a ≥ 2-fold increase of cytokine+ CD4 T cells and anti-H56 IgG levels at days 98 and/or 154 versus levels at day 84.

*Partial Responders*: patients with a ≥ 2-fold increase of cytokine+ CD4 T cells or anti-H56 IgG levels at days 98 and/or 154 versus levels at day 84.

*Non-Responders*: patients with a ≤ 2-fold increase of cytokine+ CD4 T cells and/or anti-H56 IgG levels at days 98 and/or 154 versus levels at day 84.

### RNA isolation, cDNA synthesis and preamplification

Total RNA was extracted from whole blood samples collected in PAXgene tubes using the automated PAXgene Blood miRNA Kit (PreAnalitiX, Hombrechtikon, Switzerland) procedure, according to the manufacturer’s protocol. Briefly, cells were pelleted and lysed. Cell contents were treated with proteinase K and silica-based column extraction was performed, including on-column DNAse I treatment. Total RNA quantity was determined using Qubit RNA BR Assay Kit (Thermo Fisher Scientific, Waltham, MA, USA).

cDNA was synthesized by performing reverse transcription of 50 ng RNA (incubation at 25°C for 5 minutes, 42°C for 30 minutes and 85°C for 5 minutes). Reverse Transcription Master Mix (Standard BioTools, South San Francisco, CA, USA), containing M-MLV reverse transcriptase, random hexamer, and oligo dT primers, was used. cDNA was preamplified using a pool of the target TaqMan assays (Thermo Fisher Scientific, 0.2X each in TE buffer: 10 mM Tris-HCl, pH 8.0, 0.1 mM EDTA) and Preamp Master Mix (Standard BioTools) according to manufacturer’s instructions. Thermal cycling conditions were: 95°C for 2 minutes followed by 14 cycles at 95°C for 15 seconds and 60°C for 4 minutes. Preamplified cDNA was diluted 1:5 in TE buffer and stored at -20°C prior to analysis.

### High-throughput qPCR gene expression analysis

The expression level of 183 immune-related genes (**Supplementary Tables 1**, **2**) [144 genes previously explored in other publications (22, 45, 46)] was measured by high-throughput microfluidic qPCR using 96.96 IFC chips on the Biomark HD system (Standard BioTools), as described by the manufacturer. Each TaqMan Assay (20X, FAM-MGB) was diluted in Assay Loading Reagent (Standard BioTools) to a 10x assay mix. Sample mixes were prepared containing 1x TaqMan Universal PCR Master Mix (Thermo Fisher Scientific), 1x Sample Loading Reagent (Standard BioTools) and 2.25 μl of preamplified cDNA. The 96.96 IFC chip was primed with Control Line Fluid (Standard BioTools) and assay and sample mixes were loaded into the chip using the IFC Controller HX (Standard BioTools). qPCR was performed with the Biomark HD using the following thermal cycling protocol: 95°C for 10 minutes, followed by 40 cycles at 95°C for 15 seconds and 60°C for 1 minute. Data was analysed using Standard BioTools Real-Time PCR Analysis Software (version 4.1.3). A Ct value < 35 was determined as the cut-off for reliable detection. Genes with gene expression values above the cut-off (Ct values ≥ 35) in more than 90% of the samples were removed from the analysis (*CCL11, IL5, NLRP10*, and *NLRP13)*. Relative target gene expression was determined by calculating ΔCt using *GAPDH* as a reference gene.

### Statistical analysis

Statistical analyses were performed using R software version 4.2.1 (The R Foundation for Statistical Computing, Vienna, Austria). *GAPDH*-normalized gene expression data were corrected for batch effects by using the ComBat function implemented in the R sva package 3.48.0 using the default parametric adjustment mode.

Longitudinal *Differential Expression Analysis* (DEA) was performed comparing i) H56:IC31 group versus Controls; and ii) Responders versus Controls, at all post-vaccine time points (from day 84 until day 238).

Additionally, the *Dynamics of gene expression* following vaccination was investigated. In this analysis, expression levels at day 98 were first adjusted against the pre-vaccination baseline at day 84 and then DEA was performed comparing the H56:IC31 group versus Controls.

The non-parametric Mann-Whitney U-test with Benjamini-Hochberg correction for multiple testing was applied in all comparisons. A p-value <0.05 was set as the threshold for the identification of Differential Expression Genes (DEGs). Since no DEGs were detected after multiple testing correction, we show results without the correction (p-value not adjusted). Additionally, genes showing Log2 Fold Changes (FC) <-0.6 and >0.6 were also retrieved from the analysis in order to show a more representative picture of up- and downregulated modules despite not reaching statistical significance.

To evaluate whether Responders’ expression profiles were related and different from either Partial Responders’ or Non-Responders’, Principal Components Analysis (PCA; function prcomp from R package stats) was performed at each time point. To delineate the genes contributing significantly to the first two principal components (PC1 and PC2) in our PCA model, we used the Python library sci-kit-learn. First, we extracted the Component loadings which represent the contribution of each gene to the construction of the respective principal components, serving as coefficients in the linear combinations that form PC1 and PC2; and second, a prioritization of genes based on loading magnitudes was performed allowing to pinpoint the genes that predominantly define the variance captured by each of these principal components. In addition, heatmap and clustering data analysis was performed using https://software.broadinstitute.org/morpheus. Hierarchical clustering was applied to columns and rows using correlation distance and average linkage. Normalized and batch corrected Ct values (Appendix 1) and DEA results (Appendix 2) are openly available at DOI 10.5281/zenodo.10223492.

### Analysis of the ACS-COR signature

Longitudinal expression analysis (day 0–day 238) of the genes included in the ACS-COR signature (31) (*ANKRD22, APOL1, BATF2, ETV7, FCGR1A_B_CP, GBP1, GBP2, GBP4, GBP5, SCARF1, SEPTIN4, SERPING1, STAT1, TAP1*, and *TRAFD1)*, which were among the selected set of 183 genes described above, was performed in the H56:IC31 group and Controls, and comparative signature analyses were carried out in order to explore potential differences between i) the H56:IC31 group versus Controls; and ii) Responders versus Controls. FCGR1A_B_CP expression analysis was based on the use of the probe Hs00174081_m1 (Thermo Fisher Scientific Inc.) which recognizes the Fc segment of the IgG receptor 1, making it impossible to distinguish between the expression levels of FCGR1A and FCGR1B separately, as reported in Zak et al. (31).

## Results

### Gene expression perturbations associated with adjunct H56:IC31 vaccination

**Figure 1:**
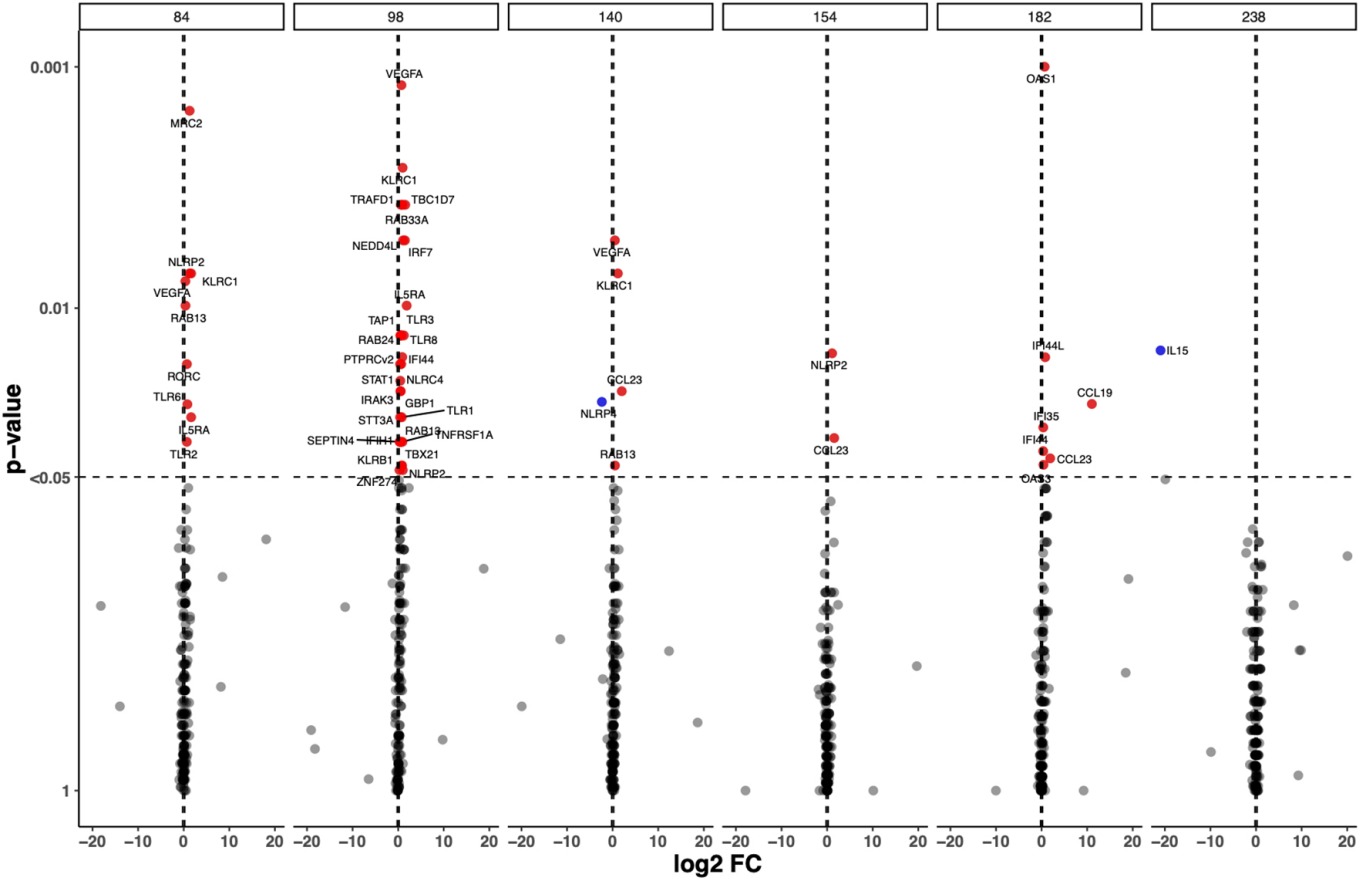
Longitudinal Differential Expressed Genes (DEGs) in the H56:IC31 group compared to Controls. Volcano plot representation of the DEGs obtained by comparing GADPH-normalized and batch effect corrected gene expression of 183 selected genes in H56:IC31 group vs Controls at day 84, 98, 140, 154, 182, and 238. Upregulated DEGs are represented with red dots and downregulated DEGs are represented using blue dots.

To analyse transcriptome perturbations in TB patients who received the adjunct H56:IC31 vaccine, we explored the longitudinal DEGs by comparing the H56:IC31 group versus Controls at five post-vaccination time points until day 238 (**Figure 1**). The most pronounced transcriptional differences between groups were observed at day 98, two weeks after the first vaccination, with 28 differentially upregulated genes (20 DEGs with a p-value < 0.05; 8 genes with a p-value <0.01) (**Figure 1**; **Supplementary Figure 1A**). These DEGs belonged to modules such as IFN signalling genes (the most perturbed module with a total of 7 genes differentially upregulated), Apoptosis and survival, Cell growth and proliferation, Cytoskeleton associated genes, E3 ubiquitin protein ligases, Inflammasome components, Inflammation, Metabolism, NK cell markers, Pattern recognition receptors, Prostanoids, Small nucleotide guanosine triphosphate (GTP)-ases, T cell subset markers, Th1-associated genes, and Transcriptional regulators (**Supplementary Figure 1A**).

**Figure 2:**
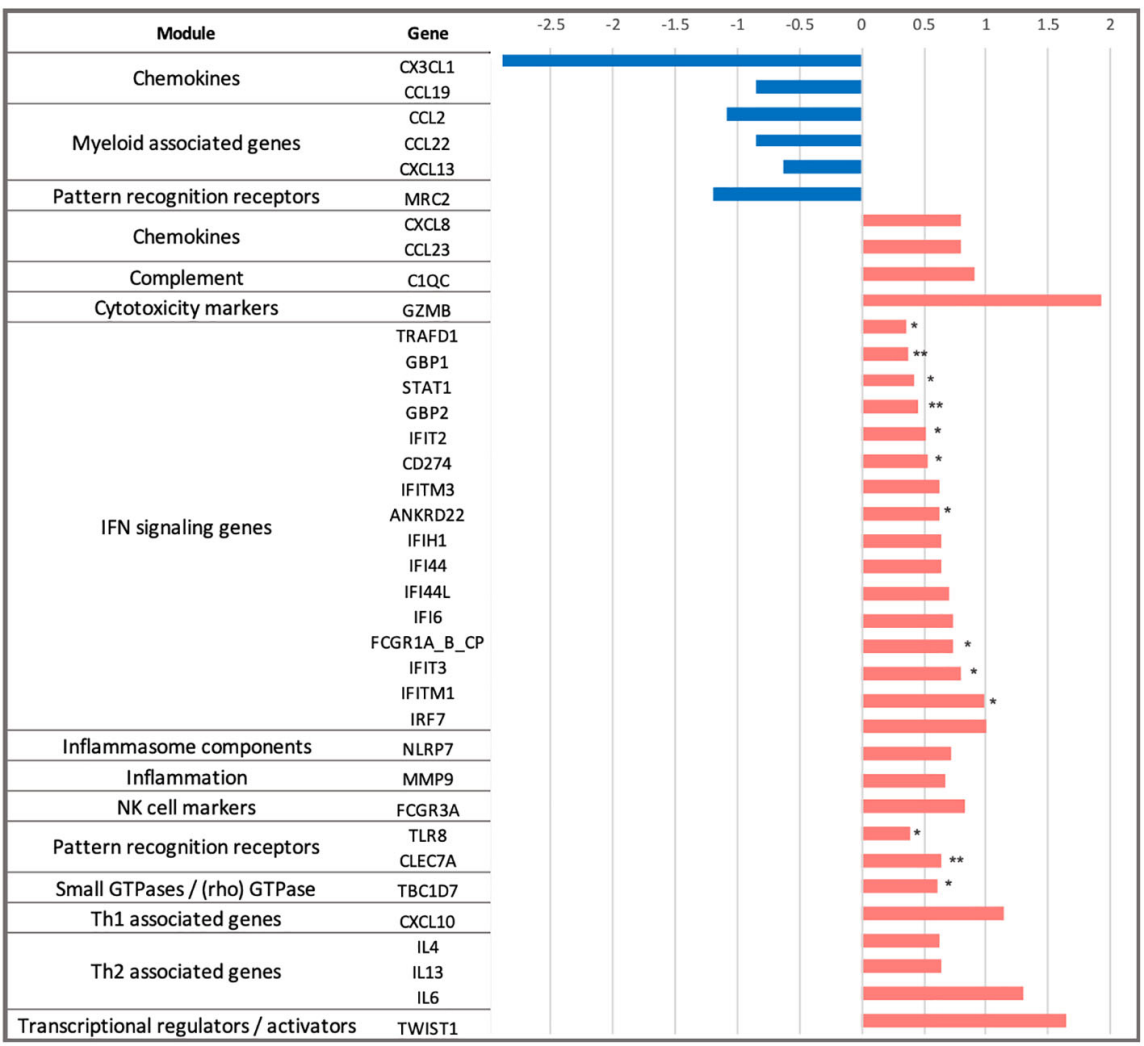
Dynamics of gene expression in the H56:IC31 group compared to Controls in the first two weeks after the first vaccination. Differential expression analysis at day 98 was baseline-adjusted (baseline at day 84). Results are represented by Log2 Fold Changes (Log2FC) between expression levels in the H56:IC31 group and Control group. Log2FC < -0.6 (depicted in blue bars) correspond to genes downregulated in the H56:IC31 group. Log2FC > 0.6 (depicted in red bars) correspond to genes upregulated. Significant differential expression levels are expressed as p-values < 0.05 (*), and <0,01 (**), between the H56:IC31 group and Control group.

Next, we explored the differences in longitudinal gene expression between the H56:IC31 group and Controls while adjusting for expression levels at the day of vaccination (day 84). At day 98, IFN signalling genes were confirmed to be the most perturbed with 10 significantly upregulated genes in the H56:IC31 group compared to Controls. Genes belonging to modules such as Patterns recognition receptors (2 genes), and small GTPases (1 gene) were also found significantly upregulated (**Figure 2**).

Different to what was observed at day 98, fewer genes were differentially expressed between the H56:IC31 group compared to Controls at days 154, 182 and 238, with only 2, 7 and 1 DEGs, respectively (**Figure 1**; **Supplementary Figure 1A**). However, considering Log2 fold change differences instead of p-values, we observed that Th1 associated genes, Chemokines, Prostanoids, NK cell markers, and Cytotoxic markers remained upregulated in the H56:IC31 group compared to Controls at day 154 (two weeks after the second vaccination) and at day 182 (end of the treatment) (**Supplementary Figure 1A**). By contrast, an opposite expression profile was observed at day 238 (two months after the end of the treatment) with the downregulation of IFN signalling genes, Pattern recognition receptors, and Th1 associated genes in the H56:IC31 group compared to Controls (**Supplementary Figure 1A**).

### H56:IC31 vaccine induced immune responses in TB disease

The H56:IC31 vaccine elicited heterogeneous humoral and T cell responses in our study cohort. An immune response was elicited in the majority of TB patients (10/11; 90.9%), of whom seven were classified as Responders, three as Partial Responders and one as a Non-Responder (**Table 1**; **Supplementary Table 3**). The H56:IC31 mediated immunogenicity was not associated to clinical or demographic characteristics of the patients (**Table 1**).

**Figure 3:**
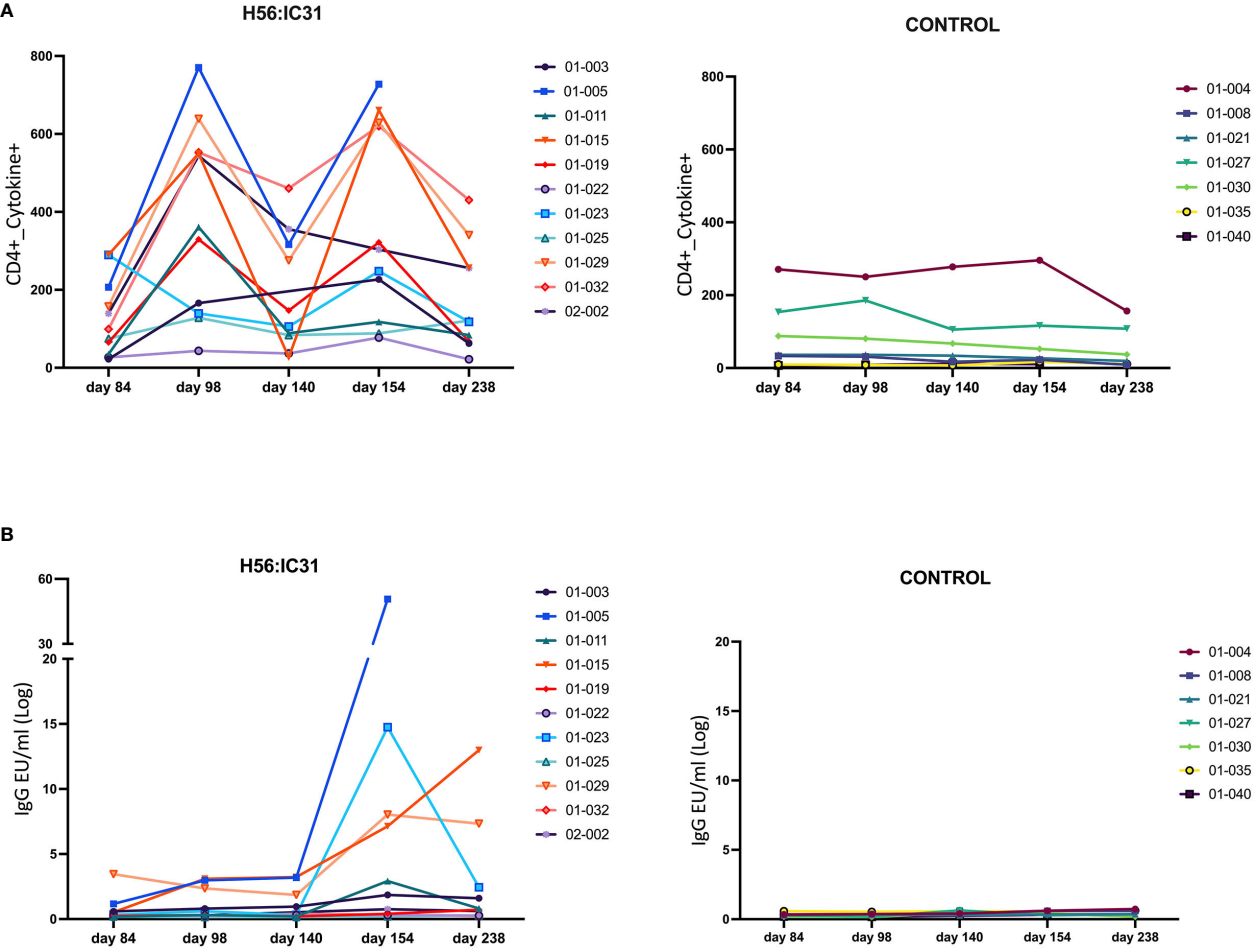
Individual and longitudinal humoral and cellular responses in the H56:IC31 and Control groups. **(A)** Individual cellular Cytokine+ CD4 T cell (sum of IFNγ and IL-2 responses to Ag85B, and ESAT-6) responses represented as Spots Forming Units (SFU)/300,000 cells, and **(B)** Log transformed anti-H56 IgG serum levels (EU/ml), from day 84 to day 238. Individual responses at all time points are represented using separate coloured linear diagrams for H56:I31 vaccinated and Control patients.

**Figure 4:**
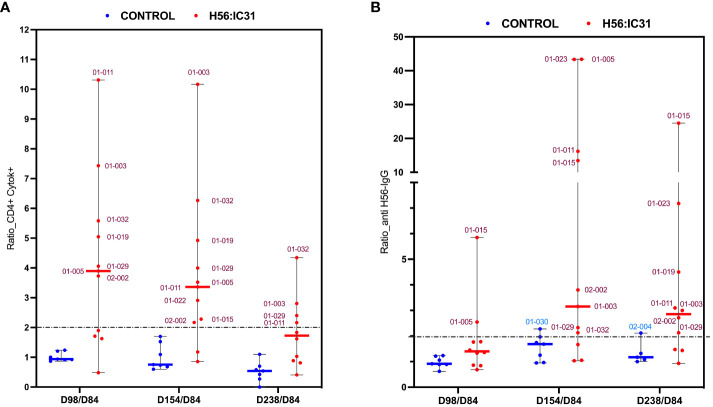
Ratios of humoral and cellular responses at different time points post-vaccination versus day 84. **(A)** Ratio of Cytokine+ CD4 T cell responses, and **(B)** ratio of log transformed anti-H56 IgG serum levels at day 98 versus day 84, day 154 versus day 84, and day 238 versus day 84, represented using boxplots with medians and range of values. H56:IC31 vaccinated patients are represented by red dots and Controls by blue dots. The cut-off established to define vaccine mediated immune responses is represented by a discontinued line at ratio equal 2. Patient’s ID are specified in the graphics for those who showed a ≥ 2-fold increase of immune responses post-vaccination.

The H56:IC31 vaccine induced a ≥ 2-fold increase of CD4 T cell responses in 7/11 patients at day 98 that remained elevated in 4/7 patients until day 238 (**Figures 3A**, **4A**). Regarding humoral responses, 2/11 patients had ≥2-fold increase in anti H56 IgG after the first vaccination (day 98), whereas additional 6 patients seroconverted after the second vaccination (day 154) (**Figures 3B**, **4B**). None of the Controls experienced ≥2-fold increase in CD4 T cell responses, but one patient had ≥2-fold increase in anti-H56 (day 154).

Since vaccine responses were heterogeneous, we first explored if gene expression profiles were associated with the levels of vaccine-elicited immunogenicity in the H56:IC31 groups. By using Principal Component Analysis (PCA), we observed Non/Partial Responders to be closely related at days 98, 140 and 182, whereas at day 238, the Non-Responder was shown as an outlier by the PC1 (**Supplementary Figure 2**). At day 98, two weeks after the first vaccine administration, PC1 distinguished most of the Responders from the Non/Partial Responders, with the genes *GZMB*, *CX3CL1, SOCS1*, and *IL9*, predominantly contributing to PC1.

However, there were no clustering of gene expression profiles associated to stratified groups according to adaptive immune responses (**Supplementary Figure 3**). Furthermore, the exclusion of Non/Partial responders in the DEA of H56:IC31 group versus Controls, did not influence our findings of early transcriptomic perturbations described for the whole group of vaccinated patients (**Supplementary Figure 1**).

### ACS-COR signature genes are downregulated in H56:IC31 vaccinated patients two months after end of TB treatment compared to controls

**Figure 5:**
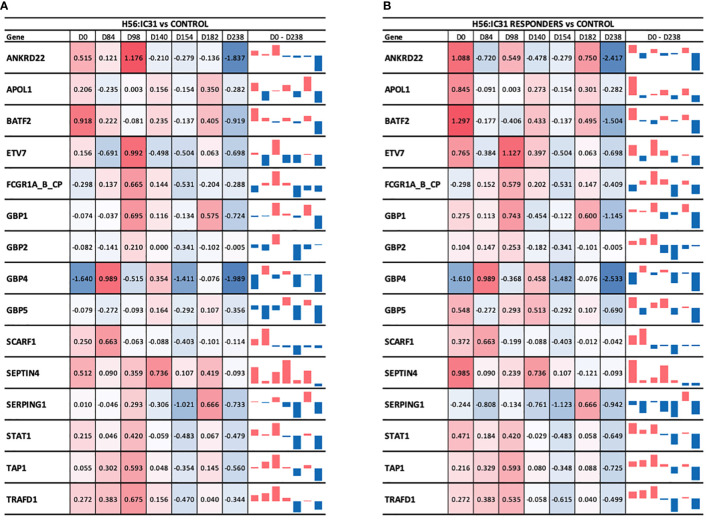
Longitudinal ACS-COR signature expression in **(A)** H56:IC31 group compared to Controls; and **(B)** Responders compared to Controls. ACS-COR genes’ expression levels in H56:IC31 group versus Controls are represented by Log2 Fold Changes. A colour scale indicates up- (red) or downregulation (blue) compared to Controls. A vertical bar representation in the last column visualizes the up or downregulation of the gene expression in vaccinated patients compared to Controls from day 0 to day 238.

At the time of TB diagnosis, the H56:IC31 group showed higher expression values of most of the ACS-COR genes compared to Controls (**Figure 5A**), corresponding to more symptomatic/advanced TB disease observed in the H56:IC31 group [**Table 1**(9)]. The longitudinal analysis of the ACS-COR signature showed downregulation of most genes towards the end of the treatment (day 182) in both the H56:IC31 group and Controls.

When comparing the evolution of the expression of ACS-COR genes between groups we observed that the expression of *ANKRD22, ETV7, GBP1, GBP2, SEPTIN4, SERPING1*, and *STAT1* was increased at day 98 in the H56:IC31 group compared to Controls despite all these genes were observed downregulated at day 84, likely as a response to standard TB treatment (**Figure 5**). By analysing the expression and dynamics of these ACS-COR genes within the H56:IC31 group, we could confirm the post-vaccination upregulation of *ETV7, GBP2*, and *STAT1* (**Supplementary Figure 4**).

Finally, we observed a “rebound” in the gene expression of the ACS-COR genes two months after end of treatment (day 238) in both study groups (**Supplementary Figure 4**), but less pronounced in the H56:IC31 group (**Supplementary Figure 4**). Notably, all the genes within the ACS-COR signature had a lower expression in the H56:IC31 group than in Controls. This observation was more prominent when only the H56:IC31 Responders were compared to the Controls (**Figure 5B**).

## Discussion

HDT is an emerging concept entailing tailored immunomodulation as part of a precision medicine treatment strategy in TB in order to improve outcomes. The goal of HDT is to increase TB cure rates by increasing Mtb control and/or reduce excessive inflammation and lung tissue destruction ultimately contributing to prevent TB relapses and Mtb reinfections (47, 48).

In the present study, we have explored transcriptomic signatures in TB patients receiving two doses of the subunit H56:IC31 vaccine adjunctive to standard TB treatment compared to Controls receiving standard treatment only. We describe differential gene expression profiles associated to immune responses elicited by the H56:IC31 vaccine, including upregulation of modules such as IFN signalling genes, pattern recognition receptors and small GTP-ases. In addition, we explored the longitudinal expression patterns of genes within the ACS-COR signature and found that most genes declined during TB treatment. Of interest, we observed that vaccinated TB patients experienced a lower expression in genes included in the ACS-COR signature two months after the end of TB treatment compared to Controls. This may indicate better infection control and reduced risk of recurrence in H56:IC31 vaccinated subjects. However, we acknowledge that the evidence of the ACS-COR signature (31) as a correlate of risk of active TB progression or recurrence did not originate from a TB treated cohort. This, together with the small sample size of our cohort, necessitates of cautious interpretations.

The upregulation of IFN signalling genes and pattern recognition receptors, essential in the initiation and shaping of adaptive immune responses, was most pronounced two weeks after the first vaccination in accordance with previous reports on transcriptional vaccine-induced perturbations (22, 26, 27, 49). Additionally, upregulated modules such as Prostanoids, Inflammation, Inflammasome components, T cell subsets and Th1-associated genes in vaccinated patients at this time point, could also reflect the IC31 adjuvant-induced inflammatory/innate immune responses pathways previously described in mouse models (50). The observed upregulation of small GTP-ases such as *RAB24, RAB13, RAB33A*, and *TBC1D7*, in vaccinated patients, might represent a novel finding associated with TB vaccination. The Rab family of small GTPases modulates immune responses by regulating the transport of immune receptors, and the secretion of chemokines and cytokines (51). They also promote immune surveillance processes such as phagocytosis, including phagosome formation and maturation, and autophagy (52, 53). Rab33A, among the small GTP-ases that we found upregulated after vaccination has been shown to be induced in T cells upon activation, and in Mtb-infected dendritic cells (54), and has been suggested a biomarker of TB disease in different settings (54, 55). Rab33A was also described in relation to rVSVΔG-ZEBOV-GP vaccination (22).

Contrary to what might be expected, transcriptomic perturbations in the H56:IC31 group were less evident at day 154, two weeks following the booster administration. Notably, patients with active TB already have a massive antigenic stimulation prior to vaccination, thus either, their adaptive immunity reached the upper limit following the first vaccination, and/or their responses to the second vaccine administration were faster, in line with previous reports (56, 57).

Ten percent of healthy individuals may fail to mount antibody responses to programmatic preventive vaccines (58). In the TBCOX2 study, H56:IC31 failed to elicit detectable immune responses in one out of eleven individuals. It is possible that TB disease has contributed to B and T cell anergy thus affecting the capacity of developing appropriate immune responses (59). This could also explain the observation of partial responses in three additional vaccinated patients.

Previous studies have reported that pre-vaccination transcriptomes might determine the extent of protective immunity after vaccination. Foureti et al. (60) described that individuals with a ‘high’ pro-inflammatory endotype before vaccination tend to elicit early innate responses, critical for CD4 T cell and plasmablast immunity with increased antibody titers. However, we failed to find specific gene expression profiles associated with the observed differences in humoral and cellular immunogenicity among individuals. There might be several reasons why we did not find any association between adaptive or pro-inflammatory responses and specific RNA signatures, particularly before vaccination, as reported by Foureti et al. Besides the heterogeneity in this rather small study cohort the transcriptomic analysis of a pre-selected pool of genes might not have provided the full map of transcriptomic responses needed to uncover associations. Most importantly, transcriptomic responses to a therapeutic vaccine in patients with TB disease may differ from expression profiles in response to preventive vaccines of healthy individuals.

Recent studies have reported PET-CT patterns consistent with active TB lesions in bacteriologically cured individuals suggesting that TB treatment does not eradicate all Mtb in all patients (61). This underlines the relevance of re-enforcing Mtb-specific immunity as a goal of HDT in TB treatment, in order to facilitate final Mtb clearance. The ACS-COR signature, constituted by a set of 16 genes, has proven to be gradually upregulated during progression towards active TB disease and symmetrically downregulated with the resolution of the infection (31). It also strongly discriminated treatment failures from cures at week 24 and significantly predicted treatment failures at week 4 (44). Recently, RISK6, a shorter signature developed through a selection of the most sensitive and discriminatory genes in the ACS-COR signature has been shown to correlate with metabolic activity in TB lung lesions observed in PET-CT, and demonstrate utility as treatment response biomarker (34, 35).

In our study, we have observed a progressive downregulation of most of the ACS-COR genes in patients within the H56:IC31 group as well as Controls, in agreement with their clinical response to TB treatment as previously reported (44). However, some of the ACS-COR genes in the H56:IC31 group increased their expression after vaccination possibly due to vaccine antigenic stimulation. Strikingly, and despite higher baseline expression levels before initiation of TB treatment (due to more symptomatic/advanced disease) as well as post-vaccination peaks in ACS-COR gene expression, patients in the H56:IC31 group, Responders in particular, had a more pronounced over-all successive downregulation of genes within the ACS-COR signature than that seen in Controls. As no microbiological or clinical treatment failures or recurrence were observed among study participants in the TBCOX2 trial, the clinical relevance of the more prominent downregulation of genes in the ACS-COR signature observed during and after treatment in H56:IC31 vaccinated TB patients warrants further examination.

There were limitations in our study. We performed transcriptional analysis of patient samples collected two weeks after immunization to align with time points for evaluation of vaccine-induced immune responses. Consequently, we acknowledge the possibility of missing the peak of early inflammatory gene responses, typically reported to occur 1-2 days post-vaccination with a subsequent decay after one week (19, 22, 23, 27). Furthermore, it remains unclear whether immunisation during the course of active TB, when there is already substantial antigenic stimulation from the ongoing infection, could have influenced the kinetics of vaccine-induced responses. We also acknowledge that other technologies such as RNA-sequencing targeting the whole transcriptome, could offer deeper insights in connection with differential vaccine responses but also complicates the identification of differences and increase the multiple-testing concern. Finally, the heterogenicity of TB patients together with the limited number of patients in our study groups restricts this study to explorative analysis with at best a hypothesis-generating potential. Comprehensive future studies are imperative to fully comprehend whether H56:IC31 responses and their dynamics correlate with the downregulation of the ACS-COR signature and with TB protection.

In conclusion, we show that the H56:IC31 vaccine when administered adjunctly to TB treatment, significantly promotes the expression of IFN signalling genes, pattern recognition receptors and small GTP-ases already following the first vaccination. The ACS-COR genes were downregulated in parallel to clinical treatment responses in both groups, but vaccinated patients, especially those who mounted both cellular and humoral vaccine responses, showed a lower expression of ACS-COR genes at two months after the end of TB treatment, which might indicate a lower risk of recurrence. Our data show that the H56:IC31 vaccine elicits favourable immune responses supporting its potential as a candidate for HDT.

## Data availability statement

The datasets presented in this study can be found in online repositories. The names of the repository/repositories and accession number(s) can be found in the article/**Supplementary Material**.

## Ethics statement

The studies involving humans were approved by Regional Ethics Committee (TBCOX2, REK SØ 2015/692), and The Norwegian Medicines Agency (EudraCT Number 2014-004986-26). The studies were conducted in accordance with the local legislation and institutional requirements. The participants provided their written informed consent to participate in this study. Written informed consent was obtained from the individual(s) for the publication of any potentially identifiable images or data included in this article.

## Author contributions

NA: Conceptualization, Data curation, Formal analysis, Investigation, Methodology, Visualization, Writing – original draft, Writing – review & editing. EV: Data curation, Formal analysis, Methodology, Software, Validation, Visualization, Writing – review & editing. SV: Data curation, Methodology, Writing – review & editing, Formal analysis. SJ: Writing – review & editing, Conceptualization, Investigation, Supervision. KT: Conceptualization, Investigation, Supervision, Writing – review & editing. RV: Writing – review & editing, Methodology. XL: Writing – review & editing, Formal analysis. RM: Conceptualization, Writing – review & editing, Funding acquisition. TO: Conceptualization, Resources, Writing – review & editing. AD: Conceptualization, Funding acquisition, Project administration, Resources, Supervision, Writing – original draft, Writing – review & editing, Investigation.
