# Supplementary material for: Whole blood RNA signatures in tuberculosis patients receiving H56:IC31 vaccine as adjunctive therapy
Source: Front Immunol. 2024 Feb 16;15:1350593. doi: 10.3389/fimmu.2024.1350593 (PMC10904528; doi:10.3389/fimmu.2024.1350593)

SUPPLEMENTAL INFORMATION

Whole blood RNA signatures in Tuberculosis patients receiving H56:IC31 vaccine as adjunctive therapy

Noelia Alonso-Rodríguez[1], Eleonora Vianello[2], Suzanne van Veen[2], Synne Jenum[1], Kristian Tonby[1,3], Rosalie van Riessen[2], Xiaoran Lai[4], Rasmus Mortensen[5], Tom H M Ottenhoff[2], Anne Ma Dyrhol-Riise[1,3]

[1]Department of Infectious Diseases, Oslo University Hospital, Oslo, Norway.
[2]Department of Infectious Diseases, Leiden University Medical Center, Leiden, The Netherlands.
[3]Institute of Clinical Medicine, University of Oslo, Oslo, Norway.
[4]Oslo Centre for Biostatistics and Epidemiology, Faculty of Medicine, University of Oslo, Oslo, Norway
[5]Dept. of Infectious Disease Immunology, Statens Serum Institut, Copenhagen, Denmark.

Table of Contents

Table 1.- List of the genes and corresponding modules included in the analysis.
Table 2.- List of primers and probes used in the high-throughput qPCR platform Biomark HD system (Standard BioTools).
Table 3.- H56:IC31 vaccine mediated responses.

Figure 1.- Post-vaccination gene expression differences in A) H56:IC31 group compared to Controls; and, B) vaccine Responders compared to Controls.
Figure 2.- Principal Component Analysis of the expression of the 183 pre-selected genes in H56:IC31 vaccinated patients at all time points.
Figure 3.- Heatmap and clustering analysis of the H56:IC31 group based on the expression of preselected genes.
Figure 4.- Evolution of the ACS-COR signature within the H56:IC31 group and Control group.

Table 1.- List of the genes and corresponding modules included in the analysis.

Module	Num. genes	Genes included per module															
**Anti-microbial activity**	2	BPI	LTF														
**Apoptosis - survival**	7	CASP8	FASLG	TNFRSF1B	BCL2	FLCN	TNFRSF1A	SCARF1									
**B cell markers**	10	CD27	PAX5	IGHD	MS4A1	CD79A	CD19	CD79B	CR2	CD38	CXCR5						
**Cell activation**	3	HCK	LYN	SLAMF7													
**Cell growth - proliferation**	6	EGF	BMP6	VEGFA	TGFBR2	AREG	TRMT2A										
**Chemokines**	7	CX3CL1	CCL19	CCL13	CXCL9	CXCL8	CCL23	CCL11									
**Complement**	4	C2	C1QA	C1QB	C1QC												
**Cytoskeleton associated gene**	3	TUBGCP6	MAP7D3	SEPTIN4													
**Cytotoxicity markers**	4	GNLY	PRF1	GZMA	GZMB												
**E3 ubiquitin protein ligases**	1	NEDD4L															
**IFN signaling genes**	29	TRAFD1	GBP1	STAT1	CD274	IFIT2	IFIH1	IFITM3	IFIT3	IFI44	IFITM1	GBP2	GBP4	TAP1	OAS1	OAS2	GBP5
		TAP2	OAS3	IFI16	IFI35	IDO1	STAT2	IFIT5	IFI6	IFI44L	ANKRD22	SOCS1	IRF7	SERPING1			
**Inflammasome components**	11	NLRP11	NLRP6	NLRP2	NLRP1	NLRC4	NLRP12	NLRP3	NLRP4	NLRP7	NLRP10	NLRP13					
**Inflammation**	8	TIFA	DSE	IRAK3	SPP1	MAP4K4	TNIP1	TIMP2	MMP9								
**Intracellular transport**	2	SEC14L1	KIF1B														
**Metabolism**	3	SDR39U1	UCP2	STT3A													
**Mitochondrial stress - proteases**	1	HPRT1															
**Myeloid associated genes**	12	CCL2	CCL22	IL12B	CXCL13	CCL5	FPR1	IL23A	CCL3	CD14	ICAM1	IL12A	CD163				
**NK cell markers**	5	KLRB1	NCAM1	KLRC1	KLRD1	FCGR3A											
**Oxidative stress**	1	APOL1															
**Pattern recognition receptors**	16	TLR8	CLEC7A	MRC2	CD209	TLR2	NOD2	MRC1	TLR7	TLR10	TLR1	TLR9	TLR4	NOD1	TLR3	TLR6	TLR5
**Prostanoids**	1	IL5RA															
**Scavenger receptors**	1	MARCO															
**Small GTPases - (rho) GTPase**	6	TBC1D7	RAB13	TAGAP	RAB33A	ASAP1	RAB24										
**T cell subset markers**	8	CD4	AIRE	CD3E	PTPRCv1	CCR7	IL7R	CD8A	PTPRCv2								
**Th1 associated genes**	7	IL15	TBX21	IL2	TNF	IL1B	IFNG	CXCL10									
**Th17 associated genes**	3	IL22RA1	RORC	IL17A													
**Th2 associated genes**	6	IL10	GATA3	IL13	IL4	IL6	IL5										
**Th9 associated genes**	1	IL9															
**Transcriptional regulators - activators**	8	ZNF331	ZNF274	ZNF532	BATF2	CAMTA1	SMARCD3	ETV7	TWIST1								
**Treg associated genes**	6	CCL4	IL2RA	LAG3	FOXP3	CTLA4	TGFB1										
**Housekeeping gene**	1	GADPH															

Table 2.- List of primers and probes used in the high-throughput qPCR platform Biomark HD system (Standard BioTools).

Gene	Assay
ABR	Hs01077828_m1
AIRE	Hs00230829_m1
ANKRD22	Hs00944018_m1
APOL1	Hs01066280_m1
AREG	Hs00950669_m1
ASAP1	Hs00987469_m1
B2M	Hs00187842_m1
BATF2	Hs00912737_m1
BCL2	Hs00608023_m1
BMP6	Hs01099594_m1
BPI	Hs01552756_m1
C1QA	Hs00381122_m1
C1QB	Hs00608019_m1
C1QC	Hs00757779_m1
C2	Hs00918862_m1
CAMTA1	Hs01051596_m1
CASP8	Hs01018151_m1
CCL11	Hs00237013_m1
CCL13	Hs00234646_m1
CCL19	Hs00171149_m1
CCL2	Hs00234140_m1
CCL22	Hs01574247_m1
CCL23	Hs00270756_m1
CCL3	Hs00234142_m1
CCL4	Hs99999148_m1
CCL5	Hs00982282_m1
CCR7	Hs01013469_m1
CD14	Hs00169122_g1
CD163	Hs00174705_m1
CD19	Hs99999192_m1
CD209	Hs01588349_m1
CD27	Hs00386811_m1
CD274	Hs00204257_m1
CD38	Hs01120071_m1
CD3E	Hs01062241_m1
CD4	Hs01058407_m1
CD79A	Hs00998119_m1
CD79B	Hs00236881_m1
CD8A	Hs00233520_m1
CLEC7A	Hs00224028_m1
CR2	Hs00153398_m1
CTLA4	Hs00175480_m1
CX3CL1	Hs00171086_m1
CXCL10	Hs00171042_m1
CXCL13	Hs00757930_m1
CXCL8	Hs00174103_m1
CXCL9	Hs00171065_m1
CXCR5	Hs00173527_m1
DSE	Hs00203441_m1
EGF	Hs01099990_m1
ETV7	Hs00903229_m1
FASLG	Hs00899442_m1
FCGR1A/B/CP	Hs00174081_m1
FCGR3A	Hs04188274_m1
FLCN	Hs00376065_m1
FOXP3	Hs01085834_m1
FPR1	Hs00181830_m1
GAPDH_g1	Hs02786624_g1
GAPDH_m1	Hs99999905_m1
GATA3	Hs00231122_m1
GBP1	Hs00977005_m1
GBP2	Hs00894837_m1
GBP4	Hs00925073_m1
GBP5	Hs00369472_m1
GNLY	Hs01120727_m1
GUSB	Hs00939627_m1
GZMA	Hs00989184_m1
GZMB	Hs00188051_m1
HCK	Hs01067403_m1
HPRT1	Hs02800695_m1
ICAM1	Hs00164932_m1
IDO1	Hs00984148_m1
IFI16	Hs00986757_m1
IFI35	Hs00413458_m1
IFI44	Hs00197427_m1
IFI44L	Hs00915292_m1
IFI6	Hs00242571_m1
IFIH1	Hs00223420_m1
IFIT2	Hs00533665_m1
IFIT3	Hs00155468_m1
IFIT5	Hs00202721_m1
IFITM1	Hs01652522_g1
IFITM3	Hs03057129_s1
IFNG	Hs00989291_m1
IGHD	Hs00378878_m1
IL10	Hs00961622_m1
IL12A	Hs01073447_m1
IL12B	Hs01011518_m1
IL13	Hs00174379_m1
IL15	Hs01003716_m1
IL17A	Hs00174383_m1
IL1B	Hs01555410_m1
IL2	Hs00174114_m1
IL22RA1	Hs00222035_m1
IL23A	Hs00372324_m1

Gene	Assay
IL2RA	Hs00907777_m1
IL4	Hs00174122_m1
IL5	Hs99999031_m1
IL5RA	Hs00602482_m1
IL6	Hs00174131_m1
IL7R	Hs00902334_m1
IL9	Hs00174125_m1
IRAK3	Hs00936103_m1
IRF7	Hs00185375_m1
KIF1B	Hs01114511_m1
KLRB1	Hs00174469_m1
KLRC1	Hs00242628_m1
KLRC2/3	Hs04192492_gH
KLRD1	Hs00233844_m1
LAG3	Hs99999160_m1
LTF	Hs00914334_m1
LYN	Hs01015816_m1
MAP4K4	Hs01101394_m1
MAP7D3	Hs00226257_m1
MARCO	Hs00198937_m1
MMP9	Hs00957562_m1
MRC1	Hs00267207_m1
MRC2	Hs00195862_m1
MS4A1	Hs00544819_m1
NCAM1	Hs00941830_m1
NEDD4L	Hs00969321_m1
NLRC4	Hs00892666_m1
NLRP1	Hs00248187_m1
NLRP10	Hs00738590_m1
NLRP11	Hs00935472_m1
NLRP12	Hs00376283_m1
NLRP13	Hs00603406_m1
NLRP2	Hs01546932_m1
NLRP3	Hs00918082_m1
NLRP4	Hs00370499_m1
NLRP6	Hs00373246_m1
NLRP7	Hs00373683_m1
NOD1	Hs01036720_m1
NOD2	Hs01550753_m1
OAS1	Hs00973635_m1
OAS2	Hs00942643_m1
OAS3	Hs00196324_m1
PAX5	Hs00277134_m1
PRF1	Hs00169473_m1
PTPRCv1	Hs04266413_m1
PTPRCv2	Hs00898488_m1
RAB13	Hs04400188_g1
RAB24	Hs01557556_g1
RAB33A	Hs00191243_m1
RORC	Hs01076112_m1
SCARF1	Hs01092477_m1
SDR39U1	Hs00220190_m1
SEC14L1	Hs01019672_m1
SEPTIN4	Hs00365352_m1
SERPING1	Hs00163781_m1
SLAMF7	Hs00904275_m1
SMARCD3	Hs00162003_m1
SOCS1	Hs00864158_g1
SPP1	Hs00959010_m1
STAT1	Hs01013996_m1
STAT2	Hs01013132_m1
STT3A	Hs00537619_m1
TAGAP	Hs00299284_m1
TAP1	Hs00388675_m1
TAP2	Hs00241060_m1
TBC1D7	Hs00964082_m1
TBX21	Hs00894392_m1
TGFB1	Hs00998133_m1
TGFBR2	Hs00234253_m1
TIFA	Hs00385268_m1
TIMP2	Hs00234278_m1
TLR1	Hs00413978_m1
TLR10	Hs01675179_m1
TLR2	Hs00152932_m1
TLR3	Hs00152933_m1
TLR4	Hs00152939_m1
TLR5	Hs00152825_m1
TLR6	Hs04975839_m1
TLR7	Hs00152971_m1
TLR8	Hs00152972_m1
TLR9	Hs00152973_m1
TNF	Hs00174128_m1
TNFRSF1A	Hs01042313_m1
TNFRSF1B	Hs00961750_m1
TNIP1	Hs00374581_m1
TNRSF18	Hs00188346_m1
TRAFD1	Hs00198630_m1
TRMT2A	Hs00224133_m1
TUBGCP6	Hs01077299_m1
TWIST1	Hs00361186_m1
UCP2	Hs01075227_m1
VEGFA	Hs00900055_m1
ZNF274	Hs00249453_m1
ZNF331	Hs00367929_m1
ZNF532	Hs00539543_m1

Color Legend

[light yellow]	MLPA genes already published
[orange]	Extra genes selected from scientific reviews
[light blue]	Extra control
[light green]	Extra B and NK cell makers
[grey]	Reference genes

Assays A

Plate 1	1	2	3	4	5	6
A	ABR	CASP8	CD274	DSE	GNLY	IFIT5
B	BCL2	CCL4	CR2	FPR1	IFI16	IL13
C	AIRE	CCL11	CD38	EGF	GUSB	IFITM1
D	BMP6	CCL5	CTLA4	GAPDH_g1	IFI35	IL15
E	ANKRD22	CCL13	CD3E	ETV7	GZMA	IFITM3
F	BPI	CCR7	CX3CL1	GAPDH_m1	IFI44	IL17A
G	APOL1	CCL19	CD4	FASLG	GZMB	IFNG
H	C1QA	CD14	CXCL10	GATA3	IFI44L	IL1B
I	AREG	CCL2	CD79A	FCGR1A/B/	HCK	IGHD
J	C1QB	CD163	CXCL13	GBP1	IFI6	IL2
K	ASAP1	CCL22	CD79B	FCGR3A	HPRT1	IL10
L	C1QC	CD19	CXCL8	GBP2	IFIH1	IL22RA1
M	B2M	CCL23	CD8A	FLCN	ICAM1	IL12A
N	C2	CD209	CXCL9	GBP4	IFIT2	IL23A
O	BATF2	CCL3	CLEC7A	FOXP3	IDO1	IL12B
P	CAMTA1	CD27	CXCR5	GBP5	IFIT3	IL2RA

Assays B

Plate 2	1	2	3	4	5	6
A	IL4	MAP4K4	NLRP3	RORC	TBC1D7	TNF
B	KIF1B	NEDD4L	OAS3	SOCS1	TLR2	TWIST1
C	IL5	MAP7D3	NLRP4	SCARF1	TBX21	TNFRSF1A
D	KLRB1	NLRC4	PAX5	SPP1	TLR3	UCP2
E	IL5RA	MARCO	NLRP6	SDR39U1	TGFB1	TNFRSF1B
F	KLRC1	NLRP1	PRF1	STAT1	TLR4	VEGFA
G	IL6	MMP9	NLRP7	SEC14L1	TGFBR2	TNIP1
H	KLRC2/3	NLRP10	PTPRCv1	STAT2	TLR5	ZNF274
I	IL7R	MRC1	NOD1	SEPTIN4	TIFA	TNRSF18
J	KLRD1	NLRP11	PTPRCv2	STT3A	TLR6	ZNF331
K	IL9	MRC2	NOD2	SERPING1	TIMP2	TRAFD1
L	LAG3	NLRP12	RAB13	TAGAP	TLR7	ZNF532
M	IRAK3	MS4A1	OAS1	SLAMF7	TLR1	TRMT2A
N	LTF	NLRP13	RAB24	TAP1	TLR8	GAPDH_g1
O	IRF7	NCAM1	OAS2	SMARCD3	TLR10	TUBGCP6
P	LYN	NLRP2	RAB33A	TAP2	TLR9	GAPDH_m1

Table 3.- H56:IC31 vaccine mediated responses. Table shows the individual Cytokine+ CD4+ T cell responses and anti-H56 IgG titers in the H56:IC31 and Control groups at days 84, 98, 140 and 154 and the ratio of responses at day 98 vs day 84, and day 154 vs day 84. CD4+ T cell responses are represented in Spots Forming Units (SFU) per 300,000 cells. Humoral responses are defined by log transformed anti-H56 IgG serum levels (EU/ml). Patients who showed a ≥ 2-fold increase in CD4+ T cell responses are highlighted in red. Patients who showed a 2-fold increase in humoral responses are highlighted in green. Responders (R); Partial Responders (PR), and Non-Responders (NR) are specified in the table.

		VACCINE											CONTROL						
		R	R	R	R	PR	PR	PR	NR	R	R	R							
IMMUNOGENICITY		01-003	01-005	01-011	01-015	01-019	01-022	01-023	01-025	01-029	01-032	02-002	01-004	01-008	01-021	01-027	01-030	01-035	01-040
Cytokine+ CD4+ T cell (SFU/300.000 cells)	D84	22.33	206.67	35.00	290.00	65.33	26.67	289.67	75.00	157.33	99.00	139.67	271.00	33.33	36.33	154.00	88.00	10.00	7.00
	D98	166.00	770.33	361.00	550.00	329.67	43.33	139.67	128.33	638.67	552.67	544.00	250.33	31.33	36.33	185.67	80.67	8.67	8.67
	D140		316.67	88.67	26.67	147.00	36.67	106.00	83.67	275.33	460.33	355.67	277.67	17.00	34.00	105.67	67.33	7.00	11.67
	D154	227	727.67	117.67	661	321.67	77.67	247.67	88.33	628.67	620.33	303.33	295.67	22.67	26.67	116.33	52.67	17.00	10.67
	RATIO D98/D84	7.43	3.73	10.31	1.90	5.05	1.62	0.48	1.71	4.06	5.58	3.89	0.92	0.94	1.00	1.21	0.92	0.87	1.24
	RATIO D154/D84	10.17	3.52	3.36	2.28	4.92	2.91	0.86	1.18	4.00	6.27	2.17	1.09	0.68	0.73	0.76	0.60	1.70	1.52
anti-H56 IgG (log EU/ml)	D84	0.59	1.17	0.18	0.53	0.24	0.29	0.34	0.19	3.45	0.16	0.2	0.35	0.3	0.28	0.29	0.18	0.58	0.19
	D98	0.8	2.98	0.32	3.1	0.32	0.25	0.6	0.16	2.36		0.29	0.37	0.37	0.34	0.18	0.16	0.53	0.17
	D140	0.96	3.19	0.2	3.23	0.2	0.31	0.32	0.16	1.86	0.41	0.53	0.41	0.38	0.28	0.64	0.47	0.58	0.24
	D154	1.86	50.7	2.92	7.13	0.4	0.3	14.75	0.2	8.03	0.34	0.76	0.61	0.59	0.35	0.28	0.41	0.55	0.32
	RATIO D98/D84	1.36	2.55	1.78	5.85	1.33	0.86	1.76	0.84	0.68		1.45	1.06	1.23	1.21	0.62	0.89	0.91	0.89
	RATIO D154/D84	3.15	43.33	16.22	13.45	1.67	1.03	43.38	1.05	2.33	2.13	3.80	1.74	1.97	1.25	0.97	2.28	0.95	1.68

Figure 1. Post-vaccination gene expression differences in A) H56:IC31 group compared to Controls; and, B) vaccine Responders compared to Controls.

Selection of genes with Log2 Fold Changes > 0.6 or < -0.6 and/or p-values lower than 0.05 (*), <0,01 (**), and <0,001 (***). Listed genes are grouped by modules at all timepoints post-vaccination (Day 98 – Day 238). A gradient of colour was applied to visualize downregulated (blue) versus upregulated (red) genes in H56:IC31vaccinated patients compared to Controls.

A)

Module	Gene	D98	D140	D154	D182	D238
Apoptosis / survival	TNFRSF1A	0.925 *	0.722			
	BCL2			0.704		0.969
	FASLG				0.744	
B cell markers	CD27	-0.700			-0.797	1.117
	IGHD	-0.618				
	MS4A1	0.638				0.657
	CXCR5	-0.617				
Cell activation	SLAMF7			0.632	0.676	
Cell growth / proliferation	VEGFA	0.711 **				
	TGFBR2	0.733				
	AREG	1.037	0.624	0.939	0.791	
Chemokines	CCL23	1.251	2.001 *	1.554 *	1.888 *	0.746
	CCL19			0.601	11.033 *	
	CX3CL1				-10.015	8.280
Complement	C1QB		0.764			
	C1QA		0.910			
	C1QC				0.753	
	C2					-0.624
Cytoskeleton associated gene	SEPTIN4	0.352 *				
	TUBGCP6		0.618			
Cytotoxicity markers	GZMA	1.319	0.835	0.821	1.097	1.176
	GZMB		1.338	0.817		
	GNLY					0.845
E3 ubiquitin protein ligases	NEDD4L	1.080 **	1.329		0.758	
IFN signaling genes	IFI44	0.888 *			0.350 *	-1.104
	IFIH1	0.365 *				
	STAT1	0.464 *				
	GBP1	0.558 *				-0.724
	TAP1	0.592 *				
	TRAFD1	0.605 **				
	IRF7	1.480 **			1.031	0.943
	IFIT2	0.603			0.622	
	FCGR1A_B_CP	0.622				
	IFIT3	0.640				
	OAS3	0.746	0.611		0.413 *	-1.315
	IDO1	0.863	1.343		1.210	-0.618
	ANKRD22	1.262				-1.837
	SOCS1	18.768	12.371	10.132	9.226	-19.904
	GBP4			-1.411		-1.989
	IFI6			0.948		-1.068
	IFI44L				0.789 *	-1.249
	IFI35				0.374 *	
	OAS1				0.661 ***	-1.113
	IFITM3					-1.082
	CD274					-0.709
	STAT2					-0.652
	SERPING1			-1.021	0.674	-0.733
Inflammasome components	NLRP11	-19.096	-11.491	-17.905	18.460	9.521
	NLRP4	-1.272	-2.364 *		-0.776	9.276
	NLRC4	0.666 *				
	NLRP2	1.020 *	1.115	1.093 *	0.846	
	NLRP12	0.723				
	NLRP7		1.104		1.083	1.012
Inflammation	IRAK3	0.487 *				
	SPP1	0.656				
	MMP9	0.794				
Metabolism	STT3A	0.428 *				
Myeloid associated genes	IL12B	-11.634	18.645	-1.614	-0.823	
	CXCL13	0.736				-0.846
	CCL3		0.663		0.944	
	CCL22		1.087			-1.333
	CCL2			1.551	0.978	
	FPR1				-0.662	
	CCL5					0.641
NK cell markers	KLRB1	0.778 *	0.910		0.649	
	KLRC1	0.974 **	1.140 **	0.948	1.269	
	FCGR3A	0.610		0.794	0.953	1.018
	KLRD1	0.806		0.663	0.664	0.720
	NCAM1	0.876				
	KLRC2_3			-0.849		1.197
Pattern recognition receptors	TLR8	0.716 *				
	TLR1	0.786 *				0.663
	TLR3	1.246 *	1.103	0.891		
	TLR4	0.777				-0.830
	TLR2	0.863				-0.778
	MRC1	1.249	0.697			-0.621
	TLR6	1.553				0.609
	MRC2		0.646			
	CD209		0.715			-2.180
	TLR9			-0.644		
	NOD2					-1.198
	CLEC7A					-0.682
Prostanoids	IL5RA	1.863 **	0.884	1.489	1.431	
Scavenger receptors	MARCO	0.677				
Small GTPases / (rho) GTPase	RAB24	0.466 *				
	RAB13	0.598 *	0.516 *			
	RAB33A	0.959 **				
	TBC1D7	1.543 **				
T cell subset markers	AIRE	-0.667		0.916		0.645
	PTPRCv2	0.425 *				
	CCR7				-0.641	0.745
	CD3E				-0.631	0.672
	CD8A					0.766
Th1 associated genes	TBX21	0.824 *	0.795	0.875	0.605	0.885
	IFNG	0.909			0.970	1.201
	IL15		-1.181	2.394	19.065	-20.945 *
	IL2		-0.601			
	CXCL10				1.197	-0.754
	TNF					-0.721
Th17 associated genes	RORC	0.856	0.642			
	IL22RA1	9.755	-19.939	-1.672	1.617	20.063
Th2 associated genes	IL4	1.350		1.012		
	IL6	2.341	1.460			-0.716
	IL10		-0.710	-0.778		
Th9 associated genes	IL9	-18.252		19.675		9.880
Transcriptional regulators / activators	TWIST1	-6.483	-2.169	-1.876	-1.185	-9.884
	ZNF274	0.293 *				
	ETV7	1.060				-0.698
	BATF2					-0.919
Treg associated genes	LAG3		0.679			1.461
	FOXP3					0.652

B)

Module	Gene	D98	D140	D154	D182	D238
Anti-microbial activity	LTF					-0.724
Apoptosis / survival	TNFRSF1A	0.870	0.685		0.724	
	BCL2			0.761		1.134 *
	FASLG				0.631	0.612
B cell markers	CD27	-0.763			-0.977	0.909
	IGHD	-0.687				0.621
	MS4A1	0.952		0.617		0.741
Cell growth / proliferation	VEGFA	0.715 **	0.513 ***			
	AREG	0.782		0.939	1.030	
	BMP6	-0.737				0.773
	EGF	-0.630				
	TGFBR2	0.867				
Chemokines	CCL23	0.929	2.092	1.407	1.419	
	CCL19			0.601	11.381 *	
	CX3CL1				-10.015	18.414
	CXCL8		0.660			
	CXCL9				0.762	
Complement	C1QA		0.988			
	C1QB		0.925	1.156		
	C1QC				0.832	
	C2		0.629			-0.624
Cytoskeleton associated gene	MAP7D3					0.618
	SEPTIN4		0.736			
Cytotoxicity markers	GNLY					1.315
	GZMA		0.676	0.605	0.662	1.344
	GZMB	-4.000	1.285	0.817	0.798	-0.655
E3 ubiquitin protein ligases	NEDD4L	1.061 *	1.421		0.938	
IFN signaling genes	ANKRD22				0.750	-2.417 *
	CD274		0.761			-0.802
	GBP1	0.743 *			0.600	-1.145
	GBP2	0.253 *				
	GBP4			-1.482		-2.533
	GBP5					-0.690
	IDO1	0.716	1.261		0.615	
	IFI44	0.767	-0.855			-1.237
	IFI44L				0.736	-1.493
	IFI6		-0.733	0.948		-1.126
	IFIH1	0.354 *				
	IFIT2	0.602			1.018	
	IFIT3				1.664	
	IFITM1		-0.838			
	IFITM3				0.804	-1.169
	IRF7	1.397 *		-1.247		
	OAS1				0.823 **	-1.131
	OAS3	0.661				-1.338
	SOCS1		9.429	-8.889	5.345	-19.904 *
	STAT1	0.420 *				-0.649
	STAT2					-0.652
	TAP1	0.593 *				-0.725
	TRAFD1	0.535 **		-0.615		
	SERPING1		-0.761	-1.123	0.666	-0.942
Inflammasome components	NLRC4	0.618				
	NLRP11	-19.096	-19.994	-17.905	19.022	19.104
	NLRP12	0.729		-0.675		
	NLRP2	1.048	1.115	0.970		
	NLRP4	-0.708	-22.072 *	-1.420	-1.195	11.379
	NLRP6		0.665			
	NLRP7	0.607	1.122			1.014
Inflammation	DSE			-0.642		
	IRAK3	0.752				
	MAP4K4			-0.753		
	MMP9					-0.926
	TIFA	0.637		-0.745		
	TIMP2					-0.605
Intracellular transport	SEC14L1	0.816	0.606		0.646	
Metabolism	STT3A	0.607 *				
	UCP2			-0.666		
Myeloid associated genes	CCL2			1.551	1.585 *	
	CCL22		1.241	-0.632		
	CCL3		0.774 *		1.079 **	
	CD14	-0.756				
	CXCL13					8.506
	ICAM1		0.431 *			
	IL12B	-3.088				10.666
	IL23A					0.799
NK cell markers	FCGR3A			0.698	0.914	1.018
	KLRB1		0.725		0.659	
	KLRC1	0.902 *	1.095 *	0.948	1.851	
	KLRC2_3	-1.064		-1.011		1.264
	KLRD1	0.786			0.839	
	NCAM1	0.711				
Pattern recognition receptors	CD209		0.664		0.691	-1.061
	CLEC7A					-0.682
	MRC1	1.687				
	MRC2	0.847	0.654			-0.668
	NOD1			-0.658		
	NOD2					-0.937
	TLR10					0.759
	TLR2	1.175				-0.778
	TLR3	1.255 *		0.602		
	TLR4	0.756				-0.943
	TLR6	1.524				
	TLR9			-0.789		
Prostanoids	IL5RA	1.653		1.489	0.930	0.856
Scavenger receptors	MARCO	0.718				
Small GTPases / (rho) GTPase	RAB13		0.606			
	RAB24	0.471 *				-0.757
	RAB33A	0.897 *				
	TBC1D7	1.476 *				
T cell subset markers	AIRE	-0.663		0.950		0.881
	CCR7				-0.752	0.910
	CD3E				-0.701	0.960
	CD8A					0.723
	IL7R	0.850				
Th1 associated genes	CXCL10		0.609		1.591	-1.540
	IFNG				1.057	1.430
	IL15			2.237	20.733	-20.945 *
	IL2					1.089
	TBX21			0.875		0.789
	TNF				0.728	
Th17 associated genes	IL22RA1	20.436	-19.939	-2.191	1.422	21.272
	RORC	0.972				
Th2 associated genes	GATA3					0.712
	IL10	-0.914		-0.878		
	IL4			1.315		-0.952
	IL6	2.402	0.807		-2.412	-1.265
Th9 associated genes	IL9	-18.252		20.181		10.564
Transcriptional regulators / activators	BATF2					-1.504
	CAMTA1	0.652				
	ETV7	1.127				-0.698
	SMARCD3			-0.680		-0.617
	TWIST1	-4.458	-2.115	-1.052	-20.102	
	ZNF274	0.338 *				
	ZNF532					0.695
Treg associated genes	CTLA4			0.857		
	FOXP3					0.725
	IL2RA					0.631
	LAG3				-0.702	1.322

Figure 2.- Principal Component Analysis of the expression of 183 pre-selected genes in H56:IC31 vaccinated patients at days 84, 98, 140, 154, 182, and 238.

We use PCA to project gene expression datasets of H56:IC31 vaccinated TB patients onto the first two components. Three H56:IC31 subgroups of patients are analysed: Non-Responders (NR) in purple, Responders (R) in orange and Partial Responders (PR) in green. The contribution of the first two principal components is given in brackets.

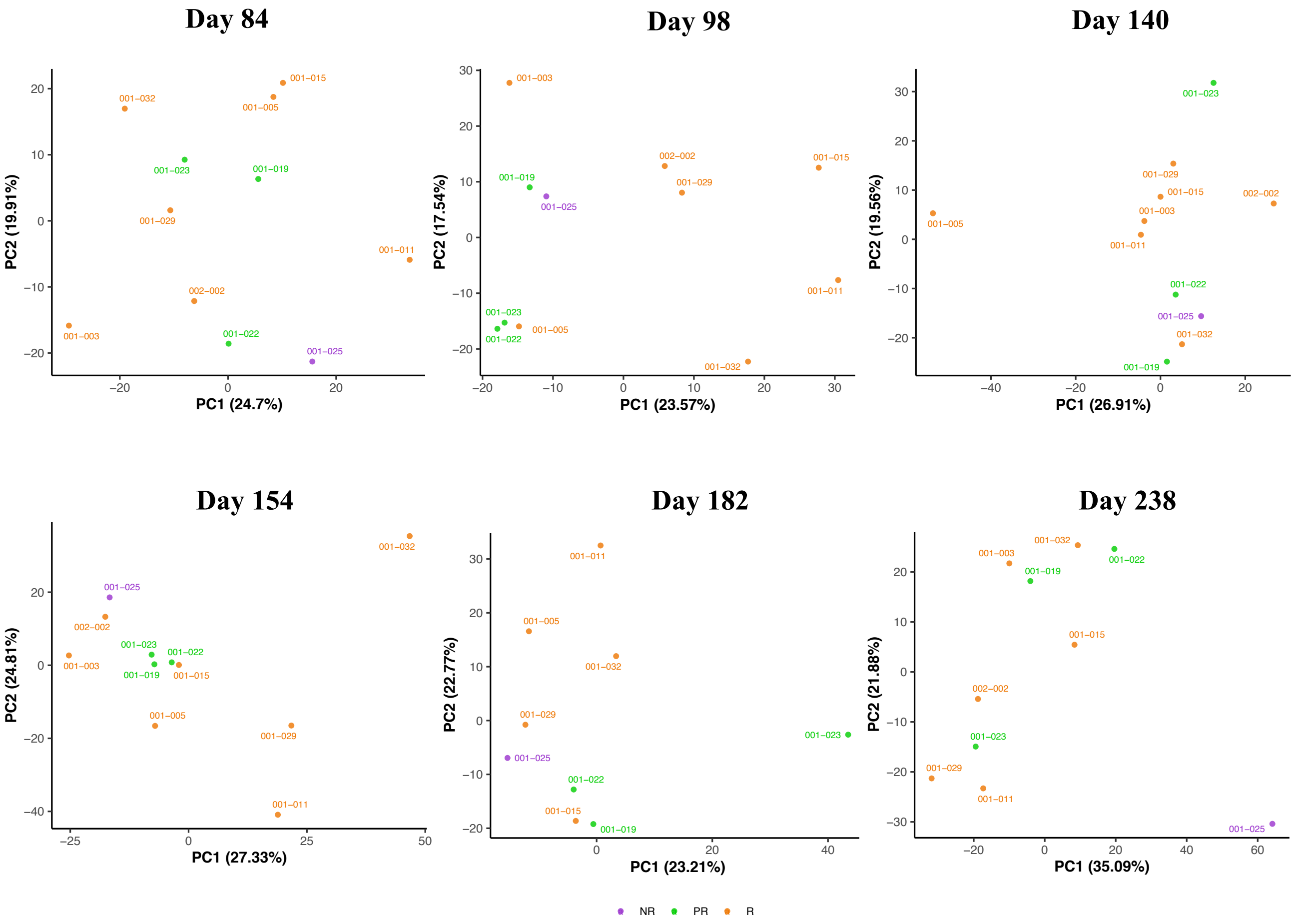

Figure 3.- Heatmap and hierarchical clustering analysis of normalized Ct values in the H56:IC31 group at A) day 84; and, B) day 98. A colour scale represent lower gene expression in blue and higher gene expression in red. Rows were centered. Both rows and columns are clustered using correlation distance and average linkage.

A)

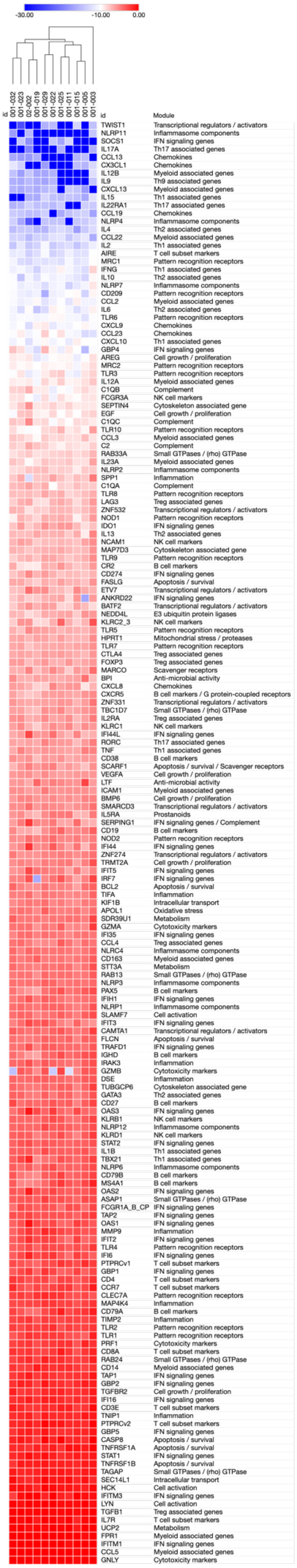

B)

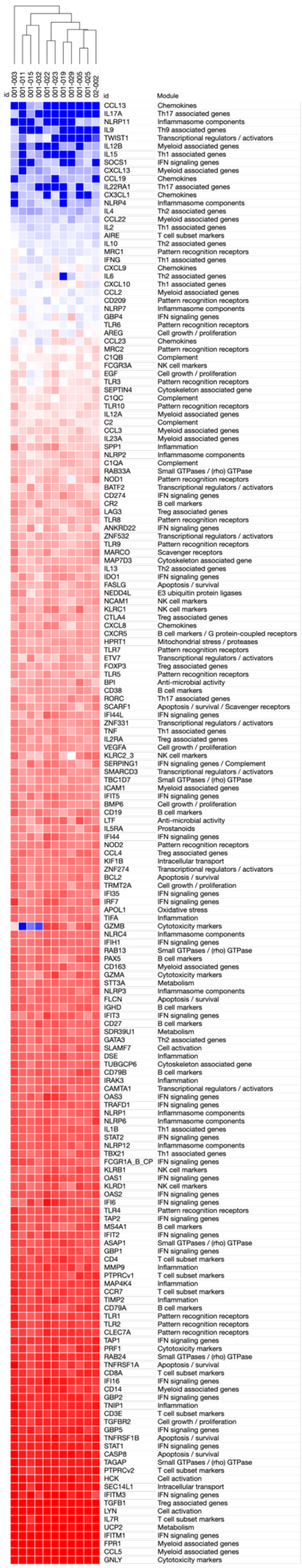

Figure 4.- Evolution of the ACS-COR signature within the H56:IC31 group and Controls. ACS-COR genes expression levels are represented as Log2FC respect the expression levels at day 0 in the H65:IC31 group (**A**) and Control group (**B**). A colour scale shows levels of upregulation (red) and downregulation (blue) at days 84, 98, 140, 154, 182 and 238, respect day 0. Blue and Red bars visually represent down and upregulated genes at the mentioned time points.

A)

H56:IC31

	Log2FC baseline D0					
**Gene**	**D84**	**D98**	**D140**	**D154**	**D182**	**D238**
**ANKRD22**	-0.554	-0.593	-0.975	-1.273	-1.023	0.070
**APOL1**	-0.286	-0.149	-0.223	-0.242	0.170	0.421
**BATF2**	-0.288	-0.779	-0.826	-1.123	-0.891	0.523
**ETV7**	-0.921	-0.343	-0.995	-0.840	-0.704	0.318
**FCGR1A_B_CP**	-0.077	-0.269	-0.768	-0.489	-0.622	0.798
**GBP1**	0.011	-0.011	-0.558	-0.592	0.006	0.372
**GBP2**	-0.116	0.084	-0.147	-0.383	-0.074	0.537
**GBP4**	1.146	0.667	0.778	-0.030	1.289	0.059
**GBP5**	-0.292	-0.303	-0.347	-0.401	-0.247	0.644
**SCARF1**	-0.029	-0.075	-0.547	-0.422	-0.200	0.159
**SEPTIN4**	-0.835	-0.737	-0.145	-0.539	-0.539	0.347
**SERPING1**	-0.287	-0.537	-1.039	-1.178	-0.407	0.242
**STAT1**	-0.166	0.000	-0.303	-0.386	-0.156	0.059
**TAP1**	0.044	0.000	-0.354	-0.392	-0.153	-0.057
**TRAFD1**	-0.117	0.336	-0.096	-0.378	0.144	0.030

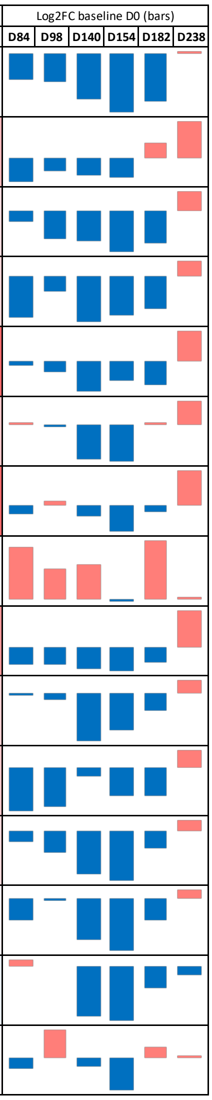

B)

CONTROL

	Log2FC baseline D0					
**Gene**	**D84**	**D98**	**D140**	**D154**	**D182**	**D238**
**ANKRD22**	-0.160	-1.253	-0.250	-0.479	-0.372	2.422
**APOL1**	0.154	0.055	-0.173	0.118	0.026	0.910
**BATF2**	0.409	0.220	-0.142	-0.068	-0.377	2.360
**ETV7**	-0.074	-1.179	-0.341	-0.181	-0.612	1.171
**FCGR1A_B_CP**	-0.512	-1.232	-1.210	-0.256	-0.716	0.788
**GBP1**	-0.026	-0.780	-0.748	-0.532	-0.642	1.022
**GBP2**	-0.056	-0.208	-0.229	-0.124	-0.053	0.460
**GBP4**	-1.483	-0.458	-1.216	-0.259	-0.275	0.408
**GBP5**	-0.099	-0.289	-0.590	-0.188	-0.432	0.922
**SCARF1**	-0.441	0.238	-0.209	0.231	0.151	0.524
**SEPTIN4**	-0.413	-0.584	-0.368	-0.135	-0.446	0.952
**SERPING1**	-0.231	-0.821	-0.723	-0.147	-1.064	0.985
**STAT1**	0.003	-0.205	-0.029	0.312	-0.009	0.752
**TAP1**	-0.203	-0.537	-0.346	0.017	-0.243	0.558
**TRAFD1**	-0.228	-0.068	0.019	0.363	0.376	0.646

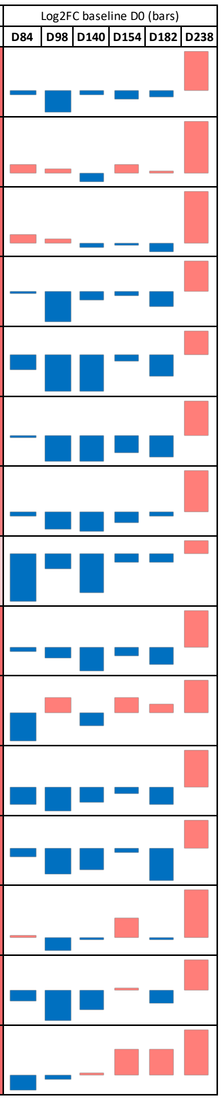

Supplement: Supplementary file 1 [file Presentation_1.pdf]
